# Is the prevalence of overweight reducing at age 5–6 years? Ten years data collection in ASL Milano 2

**DOI:** 10.1186/1824-7288-38-24

**Published:** 2012-06-08

**Authors:** Paolo Brambilla, Maria Vezzoni, Roberto Lucchini, Luigi Acerbi, Alessandra Brambilla, Giancarlo Brandolini, Patrizia Rogari, Galdino Cassavia

**Affiliations:** 1 ASL Milano 2, Melegnano, Italy; 2 Pediatric Department, University of Milano-Bicocca, Monza, Italy; 3 Via Parada 32, 20057, Vedano al Lambro, (MI), Italy

**Keywords:** Overweight, Obesity, Children, Underweight, Prevention, Blood pressure

## Abstract

**Background:**

Prevalence of overweight and obesity has been reported as high even in preschool age children. However, recent international reports suggest that prevalence is now plateauing in pediatric age. Up to now no data are available on prevalence changes in Italy in the new Millennium. Aim of the study was to describe changes of overweight and obesity prevalence during the last decade in 5–6 y children in a large Health Unit in Northern Italy.

**Methods:**

The Health Report n 8, used at 5–6 y and containing body mass index (BMI), was utilized for prevalence estimation from 2002 to 2011 according to BMI cut-offs proposed by Cole et al.

**Results:**

Overweight and obese children progressively decreased during the study period (p 0.0002) with a minimum observed in 2011, showing a cumulative frequency of 23.1% in 2002 and of 16.6% in 2011 (−6.5%). Mean BMI values progressively decreased with time so that BMI values in 2010–2011 were significantly lower than in 2002–2003 (p < 0.0001). Underweight subjects increased with time (p 0.013), from 8.2% in 2002 to 9.9% in 2011, but grade 3 underweight (i.e., severe thinness) did not increase during the study period. In years 2010 plus 2011, not Italians children showed higher percentages of underweight (12.5%) and overweight plus obesity (23.5%) respect to Italian peers (9.0% and 18.1%, respectively, p values <0.01 and 0.0029).

**Conclusions:**

This is the first report suggesting a possible decrease of overweight and obesity at 5–6 y in Italy in the last decade. As the study focused only on 5–6 y children, we don’t know if the true overweight prevalence in pediatric age is really reducing or the starting age of overweight status is simply delayed. The higher risk for malnutrition, both for excess or defect, found in our Area in not Italian children respect to Italian peers, strongly suggests to implement weight control especially for those children. Our finding needs further confirm studies but seems encouraging for true prevention of such condition.

## Background

Childhood obesity has been described as the main health-related problem in developed countries, due to its link with physical, social and psychological consequences [1]. In Italy, at age 2–6 years prevalence of overweight and obesity has reached percentages similar to those of following years [2]. Recent reports, however, taking together data from nine countries worldwide suggest that the prevalence is now plateauing in pediatric age [3]. The stabilization of prevalence seems also confirmed in 7–9 year-old French children between 2000 and 2007 [4]. Up to now no data are available on prevalence changes in Italy in the new Millennium.

ASL Milano 2 is a Local Health Unit in which overweight and obesity prevalence surveillance at 5–6 years of age started in 2001 [5]. For this purpose the Health Report adopted by Family Pediatricians (FP) was used. The initiative was first developed by FP working in the Area and then became a Health Monitoring Project in the last years. Together with this, public awareness campaigns were conducted, as well as annual courses for FP focused on early nutrition and healthy lifestyle were performed.

The aim of the present study is to describe prevalence changes observed in the last decade in the Area for overweight and underweight at age 5–6.

## Methods

### Setting

ASL Milano 2 is a large Local Health Unit located in the southern-east suburbs of Milano (Northern Italy) whose population gradually increased from 518,557 in 2002 to 620,790 persons in 2011, mostly due to not Italians immigrants [6]. Table 1 reports the variation of main descriptors occurred in the Area during the study period. Some population data referring to 2011 are not yet available at the moment in which the present paper is written.

**Table 1:** Description of variables for Area, Children, HR8 and FP during the studied years

	**Years**				
	2002	2003	2009	2010	2011
**Area Description**					
Villages (n)	46	46	53	53	53
Inhabitants (at dec 31^st^)	518577	526023	608829	615872	620790°
NI Inhabitants (at dec 31 ^st^)	17430	22983	55183	60143	NA
**Children Description**					
Children 0–6 y living in the Area (n)	30944	31525	39248	39404	NA
NI children 0–6 y (n)	1732	2049	5740	6146	NA
NI children 0–6 y (%)	5.60	6.50	14.62	15.60	NA
**HR8 Description**					
Children 5–6 y living in the Area (n)	5040	5081	6388	6588	NA
Collected HR8 (n)	1099	1552	1033*	2765	2126
Valid HR8 (n)	585	1531	967*	2590	2117
Valid HR8/Children 5-6y living in the Area (%)	11.6	30.0	15.1*	39.3	32.1°°
Children Age (for valid HR8), y (m ± SD)	5.51 ± 0.37	5.49 ± 0.30	5.43 ± 0.31*	5.48 ± 0.32	5.52 ± 0.31
Children with known gender (n)	0	0	0	2590	2117
Males/Females				1.08	1.02
Children with known origin (n)	0	0	0	2541	2078
Not Italians/Italians				0.12	0.13
**FP Description**					
FP working in the Area (n)	81	81	88	89	89
Participant FP (n)	NA	NA	NA	74	52
Participant FP (%)	NA	NA	NA	83.1	58.4

Legenda:

*n* number.

*m ± SD* mean ± standard deviation.

*y* years.

*HR8* Health Report n 8.

*NA* Not Available.

*FP* Family Pediatricians.

*NI* Not Italian.

* data limited to the last trimester only.

° calculated at 30^th^ September 2011.

°° calculated assuming the children number of 2010.

FP started to routinely use standardized Health Reports for anthropometric data collection in children born after January 1996. Among them, the Health Report number 8 (HR8), dedicated to children aged 5.00-5.99 years, contains age, weight and height data and therefore permits to compute Body Mass Index (BMI), as weight (kg)/height (m^2^). Data from HR8 were first available in 2001 (time in which children born in 1996 reached the age of interest); HR8 were manually fulfilled. HR8 data collected in 2001–2004 were analyzed according to a specific project [5], but data concerning 2001 and 2004 were not considered due to the small number of collected HR8. Since autumn 2009 FP restarted to analyze HR8 data, now electronically collected according to an obesity-dedicated Health Monitoring Project and reporting also child’s gender, blood pressure, and waist circumference. The standardization of measurement of systolic (SBP) and diastolic (DBP) blood pressure [7] and of waist circumference (WC) [8] was specific aim of FP yearly courses yielded in the period. The ratio between WC (cm) and height (cm) (WCHr) was used in order to normalize WC variation for age and gender [9]. The calculation of the total of 5–6 y children susceptible to be evaluated by HR8 in a given year was done using the mean of children living in the Area born in two consecutive years (i.e., mean of born in 2004 and 2005 for 2010).

The number of FP working in the Area increased from 81 in 2002 to 89 in 2011. The percentages of children under 6 yrs in charge to FP respect to those living in the Area were 95% in 2003 and 89% in 2009.

### Weight and height measurements

All participant FP stated to use standardized procedures for weight and height measurements [8]. For weight they used an approved scale (90/384/EEC, SECA) with precision of 50 g and periodic calibration, weighting the child with minimal dresses and registering it with approximation of 100 g; height was measured without shoes with a stadiometer with precision of 0.1 cm, and registered with approximation of 0.5 cm. Standardization of anthropometric data collection was a topic of periodic meetings and annual courses for family pediatricians.

### Weight classes definition

The frequency of weight classes (underweight, UW; normal weight, NW; overweight, OW; and obesity, OB) was calculated according to BMI cut-off values suggested by Cole et al. [10,11]. Grade 1, 2 and 3 underweight (i.e., mild, moderate and severe thinness) were defined according to Cole et al. [11]. At age 5–6 y differences among age-gender-specific cut-offs were trivial, so that single BMI values were used, obtained as the mean value of boys and girls at age 5.5 y. Single BMI cut-off values were <13.995 for underweight, >17.325 and <19.405 for overweight, and >19.405 for obesity. A comparison between frequencies obtained using single vs six age-gender-specific BMI cut-offs (age 5.0, age 5.5, age 6.0 for both genders) was performed in 2011.

### Statistical analysis

A sample size power test, based on an expected prevalence of 25% (overweight + obesity) and a susceptible population of 6,500 children at the age of interest, estimated that the study needed to evaluate at least 30% of population each year (1950 children) in order to detect differences in the prevalence as statistically significant at an alpha level of 0.05 and with a power of 90%.

Continuous variables (age, BMI, SBP, DBP, WC, WCHr) are reported as mean ± standard deviation because of their normal distribution. Categorical variables (weight classes, gender, national origin) are reported as the number and percentage of subjects with the characteristic of interest. Between-group comparisons were performed with unpaired Student’s *T* test for continuous variables and with Fisher’s exact test for categorical variables. Comparisons among different years were done with ANOVA (continuous variables) and Chi-square test (categorical variables). Statistical analysis was performed using StatView 5.0 (SAS Institute Inc.).

## Results

Children 0–6 y living in the Area increased throughout the study period (Table 1), together with the number of not Italian (NI) children whose percentage passed from 5.3% in 2002 to 15.6% in 2010. Also the number of HR8 collected per year increased, reaching a fraction over 30% respect to the overall susceptible 5–6 y old population. Children age changed during the period of interest (p < 0.0001) but remaining very close to 5.5 y. The participation of FP was over 50%, when such information was available.

In Table 2 we reported BMI absolute values, weight categories frequencies, blood pressure, waist circumference and WCHr values observed in the study period. BMI values progressively decreased with time so that BMI in 2010–2011 was significantly lower than in 2002–2003 (p < 0.0001). BMI was never different between males and females, nor between Italians and not Italians, even if not Italians children showed a tendency towards higher BMI values (p value 0.09 and 0.26 in 2010 and 2011, respectively).

**Table 2:** BMI absolute values, weight classes frequencies, blood pressure and waist circumference values observed in the study period

	**Year**				
	**2002**	**2003**	**2009**	**2010**	**2011**
**BMI** (m ± SD)					
Subjects (n)	585	1531	967	2590	2117
all	16.36 ± 2.35	16.28 ± 1.95	16.20 ± 2.15	16.09 ± 1.97*	15.89 ± 1.81*
Males	NA	NA	NA	16.03 ± 1.88	15.91 ± 1.84
Females	NA	NA	NA	16.15 ± 2.06	15.87 ± 1.78
Italians	NA	NA	NA	16.06 ± 1.94	15.88 ± 1.76
Not Italians	NA	NA	NA	16.28 ± 2.19	16.02 ± 2.16
* p < 0.0001 vs 2002–3 (ANOVA)					
**Weight categories n (%)**					
Underweight**	48 (8.2)	111(7.3)	69 (7.2)	249 (9.2)	209 (9.9)
Normal weight	402 (68.7)	1067 (70.5)	690 (71.8)	1915 (70.7)	1555 (73.5)
Overweight	92 (15.7)	240 (15.9)	128 (13.3)	380 (14.0)	259 (12.2)
Obesity	43 (7.4)	95 (6.3)	74 (7.7)	164 (6.1)	94 (4.4)
Overweight + obesity***	135 (23.1)	335 (22.1)	202 (21.0)	544 (20.1)	353 (16.6)
* *p 0.013, *** p 0.0002 (Chi Square)					
**Blood pressure** (m ± SD)					
Subjects (n)	0	0	922	2590	1525
SBP mmHg			94.7 ± 9.9	94.7 ± 9.5	95.7 ± 9.3°
DBP mmHg			59.8 ± 8.4	60.0 ± 8.6	59.4 ± 8.9°°
° p 0.035 vs 2010, °°p < 0.001 vs 2009–2010 (ANOVA)					
**Waist circumference** (m ± SD)					
Subjects (n)	0	0	941	2590	1266
cm	NA	NA	54.7 ± 5.25	54.5 ± 4.79	53.9 ± 4.2
WCHr	NA	NA	0.484 ± 0.044	0.483 ± 0.039	0.475 ± 0.035

Legenda:

*BMI* body mass index.

*m ± SD* mean ± standard deviation.

*n* number of subjects.

*NA* Not Available.

*ANOVA* analysis of variance.

*SBP* systolic blood pressure.

*DBP* diastolic blood pressure.

*WCHr* waist circumference/height ratio.

Looking to weight categories, underweight subjects increased with time (p 0.013) passing from a nadir of 7.2% in 2009 to a maximum of 9.9% in 2011; however, the percentage of subjects with grade 3 thinness decreased from 0.85% in 2002 to 0.66% in 2011. Overweight and obese children decreased during the study period (p 0.0002) with a minimum observed in 2011, showing a cumulative frequency of 23.1% in 2002 and 16.6% in 2011 (−6.5%). The reduction of overweight plus obesity was regular, with an acceleration in the last year (Table 2). No difference was found in weight categories changes over time according to gender, while in years 2010–2011 not Italians children showed higher percentages of underweight (12.5%) and overweight plus obesity (23.5%) respect to Italian peers (9.0% and 18.1%, respectively, with p values of <0.01 and 0.0029). Concerning the comparison between single-value and six-values based BMI cut-off for weight categories classification, in 2011 we found a very low discrepancy rate (24 children out of 2117, 1.1%).

Blood pressure values, available from 2009 to 2011, showed a small but significant increase of SBP in 2011 respect to 2010 (p 0.035) and a reduction of DBP in 2011 respect to 2009–2010 (p < 0.001). Waist circumference and WCHr tended to decrease with time even if they did not reach statistical significance (p 0.40 and p 0.11, respectively).

Table 3 showed differences among weight categories for waist circumference, WCHr, and blood pressure during the period 2009–2011. Waist circumference and WCHr significantly increased through each weight category, with the highest values observed in the obese group (p < 0.0001). Systolic blood pressure was higher in obese children in 2009, and in overweight and obese children compared with normal weight ones in 2010–2011. Underweight children showed lower SBP values in both 2010 and 2011 (p < 0.0001). Diastolic blood pressure showed changes among weight categories very similar to those described for SBP (p < 0.0001).

**Table 3:** Differences among weight categories for waist circumference, WCHr, and blood pressure during the period 2009–2011

	**UW**	**NW**	**OW**	**OB**	p value
**2009**					
Waist (cm)	49.7 ± 2.9 ^a^	53.4 ± 3.3 ^b^	58.5 ± 4.1 ^c^	64.9 ± 7.0 ^d^	<0.0001
WCHr (cm/cm)	0.441 ± 0.023^a^	0.474 ± 0.031 ^b^	0.512 ± 0.034 ^c^	0.563 ± 0.062 ^d^	<0.0001
SBP (mmHg)	91.9 ± 9,7 ^a^	94.3 ± 9.5 ^a^	95.5 ± 10.0 ^a^	100.1 ± 12.3 ^b^	<0.0001
DBP (mmHg)	60.5 ± 8.7 ^a^	59.0 ± 8.1 ^a^	60.2 ± 8.5 ^a^	65.7 ± 8.6 ^b^	<0.0001
**2010**					
Waist (cm)	50.2 ± 3.0 ^a^	53.5 ± 3.3 ^b^	58.4 ± 4.0 ^c^	63.8 ± 6.2 ^d^	<0.0001
WCHr (cm/cm)	0.448 ± 0.028^a^	0.477 ± 0.029 ^b^	0.510 ± 0.033 ^c^	0.553 ± 0.052 ^d^	<0.0001
SBP (mmHg)	90.2 ± 9,1 ^a^	94.3 ± 9.0 ^b^	97.4 ± 9.4 ^c^	100.2 ± 11.2 ^d^	<0.0001
DBP (mmHg)	57.1 ± 9.1 ^a^	59.7 ± 8.3 ^b^	62.4 ± 8.0 ^c^	62.6 ± 9.8 ^c^	<0.0001
**2011**					
Waist (cm)	50.4 ± 3.0 ^a^	53.2 ± 3.1 ^b^	58.1 ± 3.9 ^c^	63.4 ± 5.6 ^d^	<0.0001
WCHr (cm/cm)	0.444 ± 0.031^a^	0.472 ± 0.028 ^b^	0.504 ± 0.033 ^c^	0.540 ± 0.051 ^d^	<0.0001
SBP (mmHg)	93.0 ± 9,5 ^a^	95.4 ± 9.3 ^b^	98.2 ± 8.3 ^c^	100.7 ± 9.5 ^c^	<0.0001
DBP (mmHg)	57.1 ± 8.8 ^a^	59.0 ± 9.0 ^b^	62.3 ± 8.2 ^c^	64.3 ± 7.9 ^c^	<0.0001

Legenda:

^a,b,c,d^ Figures not sharing the same superscript were significantly different (ANOVA).

*UW* underweight.

*NW* normal weight.

*OW* overweight.

*OB* obese.

*ANOVA* analysis of variance.

*WCHr* waist circumference/height ratio.

*SBP* systolic blood pressure.

*DBP* diastolic blood pressure.

Values are mean ± standard deviation.

## Discussion

To our knowledge, this is the first report showing a possible reduction of overweight prevalence at age 5–6 years in Italian children. Other Authors have recently observed a stabilization of prevalence in nine European and not European countries [3,4] and this phenomenon was also confirmed by similar trends found in Russia, Greenland and Scotland [12-14]. In all these reports, only speculations were done about the reasons for this change respect to previous years steeply increase. A recent systematic review supported an overall levelling off of the epidemic in children and adolescents from Australia, Europe, Japan and the USA, and the levelling off was less evident in the lower socioeconomic status groups [15].

In our Local Health Unit, a surveillance of BMI at age 5–6 y, even if discontinuous, started since 2001 and allows us to state a progressive reduction of both overweight and obesity at this age thanks to the use of the same monitoring tool, such the HR8 is. Progressively with years this tool was enriched of new information (i.e., blood pressure and waist circumference), but when we simply looked to BMI data we were able to describe changes during the entire last decade. The entity of prevalence reduction of overweight together with obesity (from 23.1 to 16.6%), really surprising, needs to be confirmed in the next years, but the direction of the observed change seems sufficiently clear to us. Moreover, the actual prevalence is lower than that reported by other studies in Northern Italy at similar age [2].

When considering the reasons responsible for this, our study can only add some speculations, taking into account that it did not include food intake, lifestyle or socioeconomic status markers. We can only state that in the meanwhile some public awareness campaigns were conducted, as well as yearly obesity prevention courses and workshops for all FP working in the Area, based on early nutrition and healthy lifestyle. The study focused only on 5–6 y children so that we don’t know if the true overweight prevalence in pediatric age is really reducing or, on the contrary, the starting age is only delayed. Moreover, our results should be strictly referred to our Area, as no data on what it is happening in other Italian regions, even with higher prevalence rates respect to ours, are available. Also changes occurred in our Local Health Unit could have influenced the results. Children 0–6 y living in the Area increased throughout the study period, together with the number of not Italian children whose percentage passed from 5.3% in 2002 to 15.6% in 2010. However, the analysis of differences between Italians vs. non Italians children, available only in the last few years, seems to indicate that the latter may have a higher risk for both underweight and overweight. It has to be mentioned that a major US Surveillance System based on low income children has shown a stabilization in obesity levels in such subgroup which is probably formed by a high rate of immigrants [16]. The higher risk for malnutrition, both in excess or defect, found in our Area in non Italians strongly suggests to implement weight control especially for those children.

It has to be underlined the overall increase of underweight children over time, from 8.2% in 2002 to 9.9% in 2011, that should be examined with extreme care, taking into account the paucity of information in literature on this topic. However, the number of subjects with severe underweight decreased during the study period, suggesting that a real concern about clinical impact of this condition is limited to a very small percentage (<1%) of children.

Our study did not allow information concerning gender until 2010 so we were compelled to use gender-independent BMI cut-offs in order to define weight categories, obtained from the mean of BMI values proposed for both genders at age 5.5. However, when both methods were compared, we found a very small discrepancy in the classification rate, suggesting that this point should not have affected our findings. Also, when available, gender did not influence BMI values, very similar between males and females, supporting our choice of a single BMI cut-off at this age where BMI normative curves are flat and very close between genders [11,12].

Data on blood pressure and waist circumference were limited to the last three years and therefore we could not analyze variations of these parameters over time. However, both blood pressure and waist circumference data showed a clear cross-sectional relationship with weight categories with a significant increase of both parameters in overweight and obese children, as previously described [17,18]. It seems encouraging, however, that waist-to-height ratio might show a trend towards a reduction with time associated with overweight prevalence decrease. This fact should be important, if confirmed in the next years, as waist-to-height ratio demonstrated to be an important risk factor for obesity-related health consequences [19,20].

A limitation of the study is the lack of information concerning eating patterns and lifestyle habits changes, as well as socioeconomic status markers variations occurred during the study period, which could be related with the described BMI changes. Another limitation is that we did not evaluate the entire population of 5–6 y children living in the Area. However, we note that the previously estimated threshold of 30 % was reached in the majority of years during the study period and therefore results should be solid even from a statistical point of view.

## Conclusions

In conclusion, our study describes a reduction of both overweight and obesity at age 5–6 years in our Local Health Unit during the last decade, suggesting a possible reduction or at least a delay of overweight starting age. This finding, together with a possible slight increase of underweight, needs further confirm studies in future but seems encouraging for true prevention of such condition.

## Abbreviations

HR8, Health Report n 8; NHBPEP, National Health Blood Pressure Education Program; SBP, Systolic blood pressure; DBP, Diastolic blood pressure; WCHr, Waist circumference/height ratio; FP, Family Pediatricians; BMI, Body mass index; UW, Underweight children; NW, Normal weight children; OW, Overweight children; OB, Obese children.

## Competing interests

Authors declare that they have no competing interests.

## Authors’ contribution

PB principal investigator, design, data collection and analysis, article preparation; MV design, data collection, article preparation; RL, LA and GC data collection and article preparation; AB data analysis and article preparation; GB and PR data collection, annual courses organization, and article preparation. All authors read and approved the final manuscript.
